# Evaluation of cell-free DNA from papanicolaou smears and peripheral blood to detect endometrial cancer

**DOI:** 10.3389/fonc.2025.1570938

**Published:** 2025-08-11

**Authors:** Yao Wang, Jia-Xin Yang, Mei Yu

**Affiliations:** ^1^ Department of Obstetrics and Gynecology, China-Japan Friendship Hospital, Beijing, China; ^2^ Department of Obstetrics and Gynecology, Peking Union Medical College Hospital, Chinese Academy of Medical Science and Peking Union Medical College, Beijing, China

**Keywords:** endometrial cancer, endometrial intraepithelial neoplasia, liquid biopsy, cell-free DNA, cSMART

## Abstract

**Objective:**

This study aimed to identify tumor-derived DNA in Papanicolaou (Pap) smears and plasma specimens from patients with endometrial cancer or endometrial intraepithelial neoplasia (EC/EIN).

**Methods:**

Tumor tissue, peripheral blood, and Pap smear samples were collected from 84 patients with EC and EIN. Somatic mutations in tumor specimens were analyzed using targeted exome sequencing with a 363-cancer-related gene panel. Circulating single-molecule amplification and resequencing technology (cSMART) was used to evaluate somatic mutations in Pap smear and plasma circulating cell-free DNA (cfDNA).

**Results:**

Higher pathological grades and lymph node metastases in EC were associated with elevated plasma cfDNA concentrations (*p* < 0.05 for both). Mutations corresponding to tissue samples were identified in 42.9% of plasma cfDNA and 77.4% of Pap smear cfDNA, with Pap smears demonstrating a higher detection rate (*p* < 0.05). In patients with EC, the detection rate of consistent mutations in cfDNA from peripheral blood was significantly elevated in those with higher pathological grades, lymphovascular space involvement, and lymph node metastasis (p < 0.05). The detection rate of consistent mutations in cfDNA from Pap smears was significantly higher in the EC group (*p* < 0.001).

**Conclusion:**

Multi-gene panels can detect tumor-derived DNA in cfDNA from both blood and Pap smears of patients with EC, with Pap smear cfDNA potentially offering higher efficacy for liquid biopsies in EC. This approach could complement tissue biopsies for early diagnosis and risk stratification, warranting further investigation into the clinical utility of liquid biopsies for EC management.

## 1 Introduction

Endometrial cancer (EC) is one of the most common female reproductive malignancies (1). In developed countries and some economically developed cities in China, the incidence rate is higher than that of other gynecological malignancies (2). According to the latest national cancer registry data released by the National Cancer Center of China (NCC), endometrial cancer accounted for 77,000 incident cases and 13,500 cancer-attributed deaths in 2022, corresponding to a crude incidence rate of 7.03 per 100,000 women and a mortality rate of 1.06 per 100,000 (3). Generally, EC has a high cure rate and good prognosis. However, the mortality rate of EC has increased annually, which may be attributed to the advanced stage at diagnosis and poor differentiation (1, 4). Up to 30% of patients with EC are primarily diagnosed with stage III or IV disease and have poor outcomes. For patients in the early stage, surgery alone can result in a better prognosis, whereas for patients in the advanced stage, comprehensive treatment that includes surgery, chemotherapy, radiotherapy, targeted therapy, or immunotherapy may be required. Therefore, early diagnosis is critical for improving the survival rate and prognosis of patients with EC.

Currently, the diagnostic methods for EC that are commonly used in clinical practice include transvaginal ultrasound, plasma cancer antigen 125 (CA125) detection, diagnostic curettage, and hysteroscopy. However, each of these examinations has certain limitations that may affect their diagnostic accuracy. Precision medicine has become a prominent focus in medical advancements, and the search for a highly sensitive, specific, and noninvasive diagnostic method for the early detection of EC is of considerable importance. Liquid biopsy, an emerging noninvasive detection technology, has received increasing attention owing to its advantages of easy accessibility, noninvasiveness, and reproducibility (5). Genetic information regarding tumors can be acquired by detecting molecular markers in the body fluids of patients. Tumor-derived DNA is present in the peripheral blood and Papanicolaou (Pap) smears of patients with EC and contains tumor-related genetic information (6). Detection of tumor-derived DNA and related genetic mutations from Pap smears and peripheral blood using highly sensitive DNA detection technology can help diagnose tumor types in patients, dynamically monitor disease burden, and select optimal treatment regimens.

## 2 Materials and methods

### 2.1 Patient selection

Between September 2017 and September 2018, patients undergoing treatment for newly diagnosed endometrial cancer or endometrial intraepithelial neoplasia were enrolled in a study approved by the Ethics Committee of Peking Union Medical College Hospital (PUMCH), Beijing, China (HS-1704). Written informed consent was obtained from all participants of this study at admission to PUMCH. Patients who were highly suspected of having invasive cervical cancer, a precancerous lesion, or human papillomavirus (HPV) infection were excluded. Tumor staging and grading were performed according to International Federation of Gynecology and Obstetrics (FIGO) 2009 standards.

### 2.2 Sample collection and processing

Fresh tumor tissue was collected from the primary carcinoma site during the operation. Cervical cells, naturally shed from the cervix, were collected using Pap smears and a liquid-based cytology method. Peripheral blood from patients was collected in a cell-free (CF) tube prepared with a special material containing a protective agent. The blood was allowed to stand at room temperature (15–25°C) for 1 h, and the protective agent was fully reacted; the sample was then stored at 4°C. Blood samples were processed within 24 h of collection. The blood was separated by centrifugation at 1,600 × g for 10 min at 4°C, and the plasma was again centrifuged at 16,000 × g for 10 min to remove impurities to obtain fresh plasma.

### 2.3 Extraction and quantification of genomic DNA and cfDNA

Genomic DNA was extracted from tumor tissues and sloughed-off cervical cells using the Qiagen DNeasy Blood & Tissue Kit with a mini-spin column, following the manufacturer’s instructions.

Peripheral leukocyte gDNA and cfDNA were extracted using magnetic beads. Leukocyte gDNA extraction was carried out using an AxyPrep Mag Tissue-Blood gDNA Kit (Axygen), following the manufacturer’s instructions.

cfDNA extraction was performed with a MagMAX Cell-Free DNA Isolation Kit, following the manufacturer’s instructions.

All DNA samples were purified, and quantified with a Qubit Fluorometer and the Qubit dsDNA HS Assay Kit (Invitrogen). All gDNA, except cfDNA, was required to be fragmented.

### 2.4 Next-generation sequencing library construction and somatic variant analysis

Tumor and matched leukocyte genomic DNA were processed using the xGen Pan-Cancer Panel (IDT; 7,816 probes targeting 800 kb genomic regions) for hybrid capture-based library preparation, incorporating universal adaptor ligation, PCR amplification, and blocking oligonucleotides to minimize off-target binding (7).Libraries were sequenced on Illumina HiSeq 2500 with 2×150 bp paired-end reads.

After the sequencing, the FASTQ file was used for alignment and variant calling. To filter poor-quality reads, flexbar V2.4 software was used to process the raw read data files by removing the sequence of the original reads data and low-quality sequenced bases. The retained sequencing reads were aligned to the reference human genome (NCBI Human Genome Build 37, hg19) using the Burrows-Wheeler Aligner (BWA, version 0.5.9) software. SAMtools (Sequence Alignment/Map tools, version 1.57) was used to integrate the alignment information. The Genome Analysis Toolkit (GATK, version 3.6), a widely used tool for genetic variant discovery, was applied to identify single nucleotide variants (SNVs) and insertions/deletions (INDELs) based on the alignment results. Additional databases and tools were used to annotate the identified genetic variants, including Variant Tools (version 3.0, https://vatlab.github.io/vat-docs/), ANNOVAR (version 3.5a, http://annovar.openbioinformatics.org/en/latest/), the dbSNP database (https://www.ncbi.nlm.nih.gov/snp/), ClinVar (https://www.ncbi.nlm.nih.gov/clinvar/), and COSMIC (Catalogue Of Somatic Mutations In Cancer, version 86, https://cancer.sanger.ac.uk/cosmic/).

### 2.5 cSMART

This study utilized the cSMART platform for library construction, demonstrating exceptional performance: achieving mean sequencing depth of 40,000× with >90% uniformity (hotspots covered at >20,000×), detecting low-frequency variants at 0.01% (1 in 10,000 positives), and showing high concordance with >90% positive predictive value, >99% negative predictive value, and >95% overall agreement (8). Using established multiplex cSMART protocols (6, 8), we quantified mutations in cfDNA and Pap smear samples by counting uniquely barcoded single allelic molecules.

### 2.6 Statistical analysis

Statistical analysis was performed using SPSS Statistics ver. 24 (IBM Corp., Armonk, NY, USA). The mean and median values were compared using Student’s t-test or Mann-Whitney U test after the Kolmogorov-Smirnov test for evaluating the normality of distribution of variables. Frequency distribution was compared using chi-squared test; however, if the expected frequency was less than 5, the Fisher’s exact test was used. All *p* values were two-sided, and *p* < 0.05 was considered statistically significant.

## 3 Results

### 3.1 Patients and samples

During the study period, 186 patients with suspected EC or endometrial intraepithelial neoplasia (EIN) were enrolled. The patients were fully informed of the risks and benefits associated with participation in the study. At enrollment, the patients were advised that all sample collections would be conducted without compromising their diagnosis or treatment. For example, tumor tissue sampling was performed to ensure sufficient material for the pathological examination. Pap smears were obtained from residual cells following cervical cytology assessments. Consequently, obtaining tumor tissues from small lesions or those that are not visible to the naked eye, such as certain ECs or atypical hyperplasia, was challenging.

**Figure 1:**
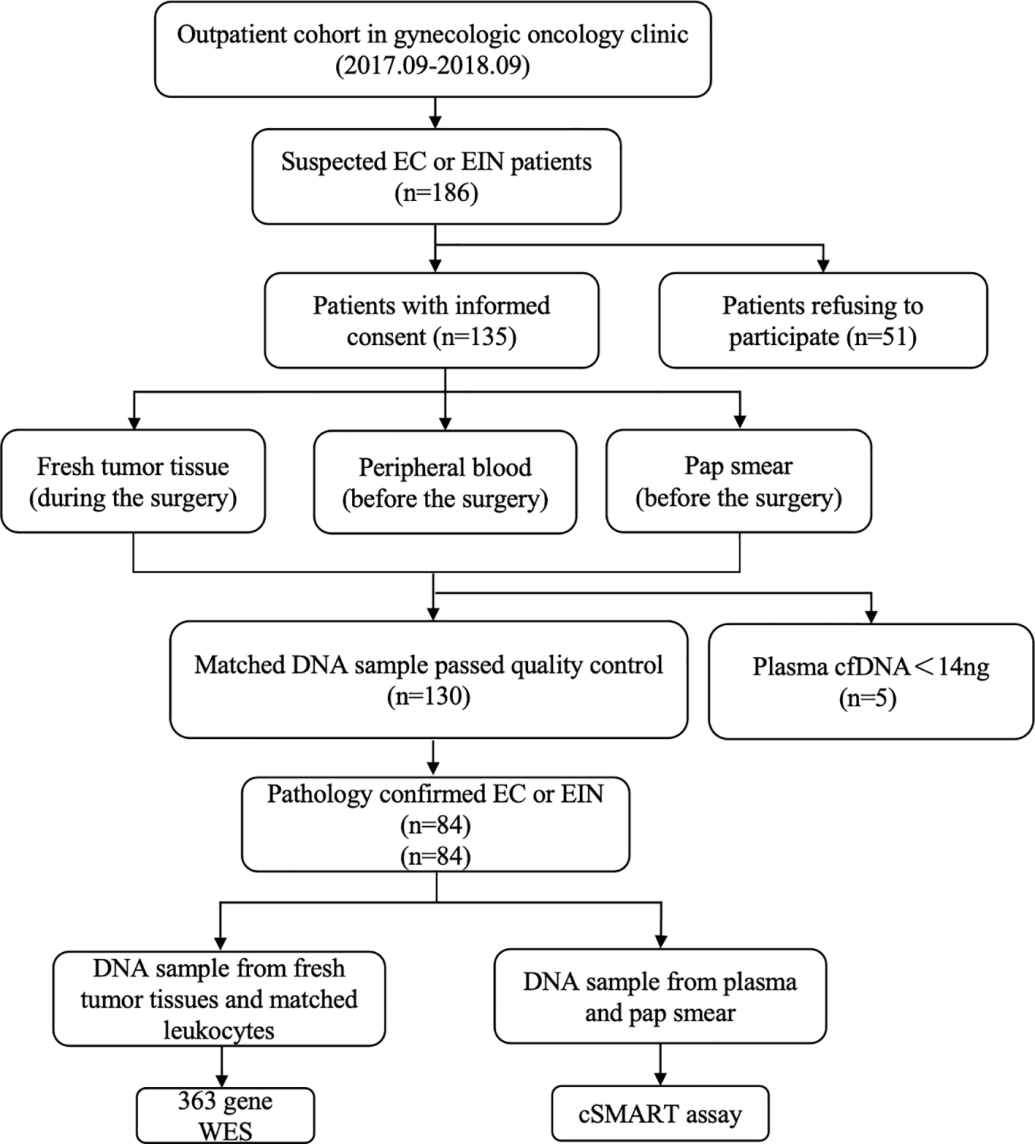
Patient enrollment, sample collection, and analysis workflow. Between September 2017 and September 2018, 186 suspected EC/EIN patients were identified in a gynecologic oncology clinic. After informed consent (n=135), matched preoperative samples (peripheral blood, Pap smear) and intraoperative tumor tissues were collected. Qualified DNA samples were obtained for 130 patients, with 5 excluded due to low plasma cfDNA (<14 ng). Pathology confirmed EC/EIN in 84 cases. Tumor/leukocyte DNA underwent 363-gene WES, while plasma cfDNA and Pap smear DNA were analyzed by cSMART assay.

Of the 186 patients, 135 consented to participate in the study after the pathological confirmation of EC or EIN. Fresh tumor specimens were procured during surgical procedures, and peripheral blood and Pap smear samples were collected preoperatively. Of the 130 patients whose matched DNA samples passed the quality control, 84 had pathologically confirmed EC or EIN (**Figure 1**). The clinical and pathological characteristics of the 84 patients (58 with EC and 26 with EIN) are summarized in **Table 1**.

**Table 1:** Clinical characteristics of patients.

Characteristics	*Values / Frequency (%)*
Age	47.5 (26-80)
Body mass index (kg/m2)	25.9 (17.6-45.5)
<24	47 (56.0%)
≥24	37 (44.0%)
CA125 (U/mL)	17.5 (6.4-1555.0)
<35	43 (51.2%)
≥35	41 (48.8%)
Plasma cfDNA (ng/ml)	6.8 (3.0-44.7)
Pap smear DNA (ng/ml)	19.2 (1.54-32.2)
EIN	26 (31.0%)
EC histology	58 (69.0%)
G1	26 (44.8%)
G2-G3	32 (55.2%)
EC FIGO	58 (69.0%)
I-II	44 (52.4%)
III-IV	14 (16.7%)
Pap smear	
Atypical glandular cells/adenocarcinoma	3 (3.6%)
Other	81 (96.4%)

CA125, cancer antigen 125; EIN, endometrial intraepithelial neoplasia; EC, endometrial cancer; FIGO, International Federation of Gynecology and Obstetrics.

### 3.2 Concentration of circulating free DNA in body fluids

The median concentration of cell-free DNA (cfDNA) extracted from plasma samples was 6.8 ng/ml (range: 3.0–44.7 ng/ml). In the EIN and EC groups, the median cfDNA concentrations were 5.7 ng/ml (range: 3.1–15.5 ng/ml) and 7.0 ng/ml (range: 3.0–44.7 ng/ml), respectively. The difference in cfDNA concentration between the two groups was not statistically significant (*p* = 0.50). In the EC group, patients with higher histopathological grades (G2–3) exhibited significantly higher cfDNA concentrations than those with lower histopathological grades (G1) (*p* = 0.004). Additionally, the cfDNA concentration was higher in the lymph node metastasis group than in the non-metastatic group (*p* = 0.03). Although the cfDNA concentration in the peripheral blood tended to be higher in patients with Federation of Gynecology and Obstetrics (aO) stage II–IV and lymphovascular space involvement, these differences were not statistically significant (*p* = 0.06 and *p* = 0.05, respectively). No significant differences in peripheral blood cfDNA concentrations were observed in relation to varying CA125 levels or depth of myometrial invasion.

The median concentration of cfDNA derived from Pap smear samples in the EIN group was 18.6 ng/ml (range: 1.6–29.6 ng/ml), while in the EC group it was 19.9 ng/ml (range: 3.3–32.2 ng/ml). Statistical analysis revealed no significant difference in the cfDNA concentrations between the two groups (*p* = 0.08). In the EC cohort, a comprehensive analysis of potential influencing factors, including age, FIGO stage, histological grade, depth of myometrial invasion, lymphovascular space involvement, lymph node metastasis, CA125 levels, and timing of cervical smear sampling, did not identify any significant associations with the concentration of cfDNA extracted from the exfoliated cervical cell samples (**Table 2**).

**Table 2:** Relationship between DNA concentration of pap smear and clinicopathological characteristics of endometrial carcinoma (n=58).

Clinical Factor	Median DNA ng/ml (range)	*P value*
Age (year)		0.39
<50	18.6 (5.2-31.0)	
≥50	20.2 (3.3-32.2)	
FIGO stage		0.30
I-II	20.2 (3.3-32.2)	
III-IV	18.9 (6.9-28.0)	
Histological grade		0.72
G1	19.2 (8.3-31.0)	
G2-3	20.0 (3.3-32.2)	
Myometrial invasion		0.26
<1/2	20.1 (3.3-32.2)	
≥1/2	18.8 (5.2-28.0)	
LVSI		0.56
Positive	18.6 (5.2-28.0)	
Negative	22.4 (3.3-32.2)	
Lymph node metastasis		0.43
Positive	20.1 (3.3-32.2)	
Negative	18.8 (7.0-24.4)	
CA125 level (U/mL)		0.06
<35	18.6 (5.2-18.8)	
≥35	20.2 (3.3-32.2)	

FIGO, International Federation of Gynecology and Obstetrics; LVSI, lymphovascular space involvement; CA125, cancer antigen 125.

### 3.3 Detection of somatic mutations in plasma cfDNA

In the cohort of 84 patients, somatic mutations were detected in all tissue samples from the EC group (58/58,100%), whereas somatic mutations were identified in 22 samples form EIN group (22/26, 84.6%). The 12 genes with the highest mutation frequencies in tissue samples were as follows: *PTEN* (48.8%), *PIK3CA* (46.4%), *PIK3R1* (32.1%), *CTNNB1* (28.6%), *ARID1A* (19.0%), *ARID5B* (15.5%), *KRAS* (14.3%), *FGFR2* (13.1%), *KMT2D* (13.1%), *NOTCH1* (13.1%), *TP53* (11.9%), and *CTCF* (10.7%). Mutation sites in plasma cfDNA that were consistent with those in tissue were identified in 36 patients (31 with EC and 5 with EIN), encompassing 115 sites across 73 genes. The median allele mutation frequency at each site was calculated. The mutation detection rate was significantly higher in the EC group compared to the EIN group (53.4% vs 19.2%, *p*<0.001). Patients with detectable mutations in plasma cfDNA that matched tissue mutations had a median cfDNA concentration of 6.3 ng/ml (range: 3.0-44.7 ng/ml), whereas those without detectable matching mutations had a median cfDNA concentration of 7.1 ng/ml (range: 3.0-28.7 ng/ml), with no statistically significant difference between the two groups (*p*=0.86). Within the EC group, the detection rate of consistent mutation sites was elevated in patients with higher histopathological grades, lymphovascular space involvement, and lymph node metastasis, with statistically significant group differences. In contrast, FIGO stage, depth of myometrial invasion, CA125 levels, and cfDNA concentrations did not significantly impact the detection of consensus mutation sites in plasma cfDNA (**Table 3**).

**Table 3:** Relationship between gene mutation detection of plasma and clinicopathological characteristics for endometrial cancer (n=58).

Clinical factor	Plasma mutation detected n (%)	Plasma mutation not detected n (%)	*P value*
FIGO stage			0.14
I-II	21 (36.2%)	23 (39.7%)	
III-IV	10 (17.2%)	4 (6.9%)	
Histological grade			**0.01**
G1	9 (15.5%)	17 (29.3%)	
G2-3	22 (37.9%)	10 (17.2%)	
Myometrial invasion			0.23
<1/2	22 (37.9%)	23 (39.7%)	
≥1/2	9 (15.5%)	4 (6.9%)	
LVSI			**0.03**
Positive	14 (24.1%)	5 (8.6%)	
Negative	17 (29.3%)	22 (37.9%)	
Lymph node metastasis, n			**0.047**
Positive	9 (15.5%)	2 (3.4%)	
Negative	22 (37.9%)	25 (43.1%)	
CA125 value (U/ml)	18.7 (8.7-172.4)	17.6 (6.9-1555.0)	0.47
cfDNA concentration (ng/ml)	6.8 (3.0-44.7)	6.4 (3.01-28.7)	0.86

FIGO, International Federation of Gynecology and Obstetrics; LVSI, lymphovascular space involvement; CA125, cancer antigen 125.

Values in bold denote statistically significant differences between the two groups.

Among the genes included in the 16-gene panel test, which exhibit the highest mutation frequency in type I and type II EC tumor tissues (*PTEN*, *PIK3CA*, *PIK3R1*, *CTNNB1*, *ARID1A*, *ARID5B*, *KRAS*, *FGFR2*, *KMT2D*, *NOTCH1*, *CTCF*, *TP53*, *ARHGAP35*, *KDR*, *NF1*, and *FBXW7*), none of the patients with EIN exhibited mutation sites that were consistent with those in the tumor tissue. However, 28 mutation sites consistent with those in tumor tissue were identified in the plasma cfDNA of 20 patients with EC, of which five exhibited at least two mutation sites identical to those in the tissue samples. The median allelic mutation frequency detected in the plasma cfDNA was 0.08% (range: 0.03–22.60%). The genes *PTEN* (6/28), *ARID1A* (5/28), and *CTNNB1* (4/28) were found to have the highest mutation frequencies (**Table 4**). Notably, mutation sites consistent with the tissue were detected in the plasma cfDNA of five patients with type II EC.

**Table 4:** Clinical and mutation information of patients with consistent mutation sites in plasma and tissues (16-gene Panel).

Plasma number	FIGO stage	Histology	Gene	DNA variant	Protein variant	Tissue AF (%)	Plasm AF (%)
C1702031	IA	G1EEC	*PTEN*	c.388C>G	p.R130G	82.2	0.07
C1702042	IA	G3EEC	*PIK3CA*	c.1633G>A	p.E545K	44.1	0.11
C1702019	IIIB	G3EEC	*ARID1A*	c.6806C>A	p.S2269X	5.5	0.06
C1801199	IIIC	CCC	*FBXW7*	c.1436G>A	p.R479Q	32.0	0.05
C1702047	IA	G1EEC	*CTNNB1*	c.110C>T	p.S37F	21.1	0.03
C1701988	IA	G2EEC	*FBXW7*	c.1513C>T	p.R505C	46.9	0.03
C1702046	II	G3EEC	*PTEN*	c.389G>A	p.R130Q	3.5	0.11
			*KMT2D*	c.3511G>A	p.E1171K	28.7	0.06
			*NF1*	c.6787C>T	p.Q2263X	23.8	0.30
C1701955	IA	G2EEC	*PTEN*	c.377C>A	p.A126D	50.8	0.18
			*CTNNB1*	c.97T>C	p.S33P	24.8	0.06
C1702049	IIIC	G2EEC	*ARID1A*	c.5164C>T	p.R1722X	3.2	0.06
C1702005	IA	G1EEC	*PTEN*	c.389G>A	p.R130Q	7.4	0.04
C1701980	IB	G1EEC	*NF1*	c.7915C>A	p.L2639I	12.0	0.12
			*KDR*	c.2884C>T	p.R962C	12.6	0.15
C1701972	IVB	UPSC	*TP53*	c.376-1G>A	c.376-1G>A	36	0.03
C1801175	IA	CCC	*PTEN*	c.800delA	p.K267Rfs	30.4	0.08
C1800664	IVB	UPSC+G2EEC	*TP53*	c.797G>A	p.G266E	98	0.44
C1801295	IIIC	CCC + G2EEC	*KRAS*	c.35G>T	p.G12V	51.1	0.31
C1702007	IVB	G3EEC	*PIK3CA*	c.1258T>C	p.C420R	55.5	0.22
C1702018	IA	G1EEC	*CTNNB1*	c.101G>T	p.G34V	35.1	0.06
C1801186	IIIC	G2EEC	*ARID5B*	c.2989C>A	p.L997M	38.9	0.24
			*PTEN*	c.389G>A	p.R130Q	39.8	22.60
			*ARID1A*	c.6420delC	p.F2141Sfs	43.5	0.14
			*CTNNB1*	c.122C>T	p.T41I	39.8	0.04
C1801170	IIIB	G2EEC	*KRAS*	c.35G>T	p.G12V	50.3	0.17
			*ARID1A*	c.1240G>T	p.G414X	37.3	0.04
C1701975	IA	G2EEC	*ARID1A*	c.4332_4338delGCGCCGA	p.E1444Dfs	40.2	0.05

EEC, endometrioid endometrial carcinoma; CCC, clear cell carcinoma; UPSC, uterine papillary serous carcinoma; AF, allelic frequency of mutation detected.

### 3.4 Detection of somatic mutations in cfDNA from cervical pap smears

Human papillomavirus (HPV) DNA can be clinically purified from cervical scrapings. Based on this, we extracted and purified DNA from Pap smears and ascertained the presence or absence of tumor-derived DNA in the samples. A total of 84 Pap smear samples were analyzed using a 363-gene panel. Among these, 65 cases (10 EIN and 48 EC) exhibited gene mutations that were consistent with the tissue. These mutations encompassed 701 mutation sites across 235 genes, including the alleles at each mutation site. The median ratio of gene mutations was 1.34% (range: 0.03–44.30%), and the mutation detection rate in the EC group was significantly higher than that in the EIN group (48/58,82.8% vs. 10/26, 38.5%, *p* < 0.001). The median DNA concentration in patients with detectable concordant gene mutations in cfDNA from cervical exfoliated cells was 19.2 ng/ml (range: 1.5–32.2 ng/ml), whereas in patients with undetectable concordant gene mutations, the median DNA concentration was 18.3 ng/ml (range: 5.9–25.8 ng/ml). The difference between the two groups was not statistically significant (*p* = 0.16). In the EC group, no significant correlations were identified between FIGO stage, histopathological grade, depth of myometrial invasion, lymphovascular space involvement, lymph node metastasis, CA125 level at diagnosis, cfDNA concentration, and timing of cervical smear sampling in Pap smear tests (**Table 5**).

**Table 5:** Relationship between mutation detection of Pap smear and clinicopathological characteristics for endometrial cancer (n=58).

Clinical factor	Pap smear mutation detected n (%)	Pap smear mutation not detected n (%)	*P value*
FIGO stage			1.00
I-II	36 (62.1%)	8 (13.8%)	
III-IV	12 (20.7%)	2 (3.4%)	
Histological grade			0.09
G1	19 (32.8%)	7 (12.1%)	
G2-3	29 (50.0%)	3 (5.2%)	
Myometrial invasion			0.43
<1/2	36 (62.1%)	9 (15.5%)	
≥1/2	12 (20.7%)	1 (1.7%)	
LVSI			1.00
Positive	16 (27.6%)	3 (5.2%)	
Negative	32 (55.2%)	7 (12.1%)	
Lymph node metastasis			0.67
Positive	10 (17.2%)	1 (1.7%)	
Negative	38 (65.5%)	9 (15.5%)	
CA125 value (U/ml)	17.6 (8.7-1555.0)	20.5 (6.9-172.4)	0.57
cfDNA concentration (ng/ml)	19.8 (3.3-32.2)	23.2 (5.9-25.8)	0.70

FIGO, International Federation of Gynecology and Obstetrics; LVSI, lymphovascular space involvement; CA125, cancer antigen 125.

Using a 16-gene Panel, we detected consistent mutation sites with tissue in 59.5% (40 EC cases and10 EIN cases) of Pap smear samples, identifying 135 mutation sites across 50 samples, with at least two matching sites in 62.0% (31/50) of the samples. The mutation detection rate in the EC group was significantly higher than in the EIN group (69.0% vs 38.5%, *p*=0.008). Moreover, we did not detect any mutation sites in Pap smear of 10 patients with benign endometrial diseases (endometrial polyps, endometrial simple hyperplasia) that were negatively confirmed by postoperative pathology. The median allele mutation frequency detected in Pap smear was 0.75% (range: 0.05%-35.69%), genes *PTEN* (28/135), *PKI3CA* (21/135), *CTNNB1* (14/135), *KMT2D* (12/135), *ARID1A* (9/135), *NF1* (9/135) and *PIK3R1* (8/135) mutations were most frequently detected (**Tables 4**, **5** , **Supplementary Table 1**). Notably, tissue consistent mutation sites were detected in pap smear of 5 patients with type II endometrial carcinoma, and TP53 mutation was detected in 3 patients.

### 3.5 Consistent alignment of mutation sites in tissue, plasma, and pap smears

Based on the 363-gene panel, 100 identical mutation sites were detected in paired cervical smears, plasma, and tissues from 27 patients (27/84, 32.1%). Of the 115 mutation sites detected in plasma cfDNA, 87.0% (100/115) of the mutation sites were detected in cfDNA extracted from cervical smears (**Supplementary Figure 1**).

Using a 16-gene panel, we identified 28 concordant mutation sites in paired cervical smears, plasma cfDNA, and tissue samples from 18 patients (21.4%) (**Supplementary Figure 2**). Among these, *PTEN* (6/28), *ARID1A* (5/28), and *CTNNB1* (4/28) exhibited the highest mutation frequencies. A positive test result was defined as the detection of the same mutation site in both the patient’s peripheral blood and cervical smear. Among the 50 cases (10 EIN and 40 EC), 28 mutation sites were detected in the plasma, and 135 mutation sites were identified in cervical smears. Notably, all the mutation sites detected in the plasma were also found in the cervical smears.

## 4 Discussion

With the advent of next-generation sequencing (NGS) technology, an increasing number of studies have focused on the presence of tumor-derived DNA in the body fluids of patients with malignant tumors. Historically, most research on tumor-derived DNA has focused on its role in predicting tumor recurrence and guiding the selection of targeted therapeutics, with few studies examining its potential as a biomarker for the early detection of cancer. This research gap is a critical factor for the clinical application of DNA testing (9). To accurately assess the diagnostic utility of tumor-derived DNA detection in body fluids for EC, we employed cSMART, an NGS-based approach that utilizes a multi-gene panel to identify tumor-derived DNA in the plasma and Pap smear samples of patients with EC and precancerous lesions (6, 8). Our findings indicate that the multi-gene panel can detect somatic mutations associated with EC in both plasma and Pap smear samples from patients with EC lesions. Notably, tumor-derived DNA was more prevalent and readily detectable in Pap smears than in plasma samples.

The concentration of cfDNA in the plasma of healthy individuals is approximately 1–10 ng/mL, and patients with cancer exhibit elevated levels of cfDNA (10). In this study, the median cfDNA yield extracted from plasma samples was 6.8 ng/mL. Although the median cfDNA level in the EIN group was lower than that in the EC group, this difference was not statistically significant. Furthermore, higher histopathological grades and lymph node metastases were associated with significantly higher median plasma cfDNA levels, consistent with the findings of Cicchillitti et al. (11). Although plasma cfDNA concentrations tended to be higher in patients with advanced disease and lymphovascular space involvement, these trends were not statistically significant. Studies have documented that the proportion of circulating tumor DNA (ctDNA) to total cfDNA in patients with cancer varies widely, from 0.1% to 93%, and is potentially influenced by tumor type, patient-specific factors such as trauma or inflammation, detection methodologies, and the specific biomarkers selected (12, 13). Particularly in the early stages of tumorigenesis, ctDNA constitutes a very small fraction of the total cfDNA present in the plasma, with an extremely low absolute concentration, thereby presenting substantial challenges for its detection. This study employed NGS-based cSMART, which has a detection limit of 0.03% and an accuracy of 99% for ctDNA mutation detection (14). In the plasma cfDNA of 36 patients, we identified 115 mutation sites consistent with those found in the tissue samples, with a median allele mutation frequency of 0.08%.

In 2018, Bolivar et al. employed a 4-gene panel (*CTNNB1*, *PTEN*, *KRAS*, and *PIK3CA*) to analyze paired plasma and tumor tissues from 48 cases of endometrioid carcinoma. Their findings revealed that 33% of patients exhibited a mutation in the plasma that corresponded to a mutation in the tumor (15). Building on this, we aimed to expand the multi-gene panel for detecting EC by identifying high-frequency gene mutations associated with EC and its precancerous lesions in the tumor tissue. Using a 363-gene panel, we identified corresponding mutations in 42.9% of plasma samples. This detection was not influenced by cfDNA levels but was significantly correlated with histopathological grade, lymphovascular space involvement, and lymph node metastasis. Contrary to previous studies suggesting that plasma mutation-positive cases are predominantly associated with advanced disease, our study identified matching mutations in the plasma of two (2/26, 7.7%) patients with EIN and 12 (12/44, 27.3%) patients with early stage (stages I and II) EC. Our findings suggest that the presence and mutational status of circulating free DNA (cfDNA) in plasma, when evaluated alongside the clinical and pathological features of endometrial cancer (EC), may serve as a potential biomarker for EC stratification.

The most important finding of this study was the detection of somatic mutations in rare tumor cells that accumulate in the cervix after being shed from EC, using molecular assays. Clinically, it is difficult to identify EC cells in cervical cytology specimens, which is influenced by the expertise of the cytologists. The sensitivity of cervical smears in detecting EC is only 30–40%, making it difficult to distinguish cervical cancer from other benign diseases (16, 17). In this study, only three patients (5.2%) with endometrial cancer were found to have atypical glandular cells or adenocarcinomas under a microscope. Theoretically, molecular testing of DNA extracted from cervical smear samples would be more objective for assessing endometrial lesions. Tumor cells constitute a very small proportion of cervical smear cells. Based on the 363-gene panel, cSMART, with its high detection sensitivity, was used to detect the same mutation site as in the tumor tissue in 77.4% of the Pap smear specimens, and the mutation detection rate in the EC group was significantly higher than that in the EIN group. The clinicopathological features of EC and the DNA concentration in Pap smears did not affect the neoplastic mutation detection rate.

Kinde et al. used whole-exome sequencing to detect somatic mutations in –22 cases of endometrioid cancer tumor tissues and found the same mutations as those in the primary tumor, which led to the design of a 12-gene panel (*PTEN*, *TP53*, *PIK3CA*, *PPP2R1A*, *KRAS*, *NRAS*, *FBXW7*, *EGFR*, *CTNNB1*, *BRAF*, *AKT1*, and *APC*) *(* 18). Using Safe-SeqS technology with a detection sensitivity of 0.01%, they identified the same mutations in the DNA from liquid Pap smear specimens in 100% of ECs (24 of 24), which confirmed the feasibility of detecting tumor-derived DNA from Pap smears (18). Based on the 16 genes with the highest mutation frequencies in type I and II EC tissues, we developed a 16-gene panel (*PTEN*, *PIK3CA*, *PIK3R1*, *CTNNB1*, *ARID1A*, *ARID5B*, *KRAS*, *FGFR2*, *KMT2D*, *NOTCH1*, *CTCF*, *ARHGAP35*, *KDR*, *NF1*, *TP53*, and *FBXW7*), which detected the same somatic mutations in 59.5% of Pap smears and 23.8% of plasma cfDNA in the same cohort. The incidence of mutations identified in Pap smears in our study was lower than that reported by Kinde et al., which may be attributed to the large number of cases included in our study and the inclusion of a subset of EIN cases. The detection rate of somatic mutations in plasma cfDNA was lower than that of Pap smears, which may be related to the predominance of patients with EIN and early stage EC, who have lower tumor burdens. Notably, ctDNA has been shown to positively correlate with tumor burden (19). Endometrial lesions can be identified by their specific body fluid (cervical secretions), which is closer to that of the diseased organ. Therefore, the detection of tumor-derived DNA from Pap smears may be more effective than plasma for the detection of endometrial lesions. Notably, in our study, mutation sites consistent with tumor tissue were detected in the plasma and Pap smears of all patients with type II EC. However, due to the very limited number of cases (n=5), whether liquid biopsy has a broader application prospect in type II EC warrants further research.

Our study explored liquid biopsy techniques for detecting EC tumor-derived DNA in plasma and Pap smears. Previous studies have used different liquid biopsy methods to identify tumor-derived DNA in body fluids and characterize the molecular features of EC (20–22). Liquid biopsy, a noninvasive diagnostic modality, has been widely acknowledged for its potential for early cancer detection and risk stratification among predisposed populations. However, the clinical utility of liquid biopsies is not without limitations. Somatic mutations in several key driver genes, often associated with malignancies, are not exclusive to cancerous conditions. Evidence indicates that 79% (19/24) of patients with endometriosis have mutations in *ARID1A*, *KRAS*, and *PIK3CA* in the epithelial tissue of endometriotic lesions (23). These genetic mutations are also relatively prevalent in endometrial carcinomas (24). Consequently, the detection of *KRAS*, *ARID1A*, or *PIK3CA* mutations in plasma or cervical smear cfDNA does not necessarily indicate EC. Furthermore, *TP53* mutations, which are predominantly associated with non-endometrioid and poorly differentiated endometrioid carcinomas, are also found in approximately 9% of well-differentiated endometrioid carcinomas (25). Additionally, certain *TP53* mutations have been identified in the synovial tissues of patients with arthritis, highlighting the non-specificity of these genetic alterations (26, 27). Given the presence of tumor-associated gene mutations under benign conditions, extreme caution is advised when employing liquid biopsy as a tumor screening tool (28). It may be more prudent to consider liquid biopsy to monitor the recurrence of EC after hysterectomy, particularly after the initial detection of genetic mutations in the primary tumor, with subsequent surveillance of the corresponding mutations in the blood. Future prospective studies are essential to ascertain whether specific genetic mutations in the plasma can be detected years after hysterectomy and whether such mutations can serve as early biomarkers for tumor recurrence. These investigations will be instrumental in refining the role of liquid biopsies in the management of EC.

EC is characterized by a multifactorial and multistage process in which multiple genetic mutations accumulate over time. These mutations are present not only in EC but also in precancerous conditions, highlighting the importance of early detection and intervention. For high-risk groups, liquid biopsies offer a less invasive approach for monitoring the genetic changes associated with EC. The ability to detect mutations in cfDNA could significantly improve the approach to risk assessment and early intervention in these vulnerable populations. In young patients who choose to preserve their fertility, liquid biopsies could provide a valuable tool for monitoring disease progression during treatment. As some patients may experience remission, while others see their disease advance, dynamic surveillance of tumor-derived DNA mutations through liquid biopsies could offer insights into disease activity and inform treatment adjustments. Further research is needed to explore the utility of liquid biopsies for tracking EC progression and responses to treatment. This includes defining the specific mutations that serve as reliable indicators of disease status and determining the optimal frequency and timing for liquid biopsy assessment. As the field of liquid biopsy continues to evolve, it holds promise for improving our ability to predict, prevent, and manage EC, particularly among those with the highest risk.

## Data Availability

The datasets presented in this study can be found in online repositories. The names of the repository/repositories and accession number(s) can be found below: http://bigd.big.ac.cn/gsa-human/s/U6AiGcb4, HRA000033.

## Supplementary material

Supplementary Table 1 Clinical and mutation information of patients with consistent mutation sites in Pap smear and tissue (16-gene Panel). FIGO, International Federation of Gynecology and Obstetrics; EIN, endometrial intraepithelial neoplasia; EEC, endometrioid endometrial carcinoma; CCC, clear cell carcinoma; UPSC, uterine papillary serous carcinoma; AF, allelic frequency of mutation detected.

Supplementary Figure 1 Concordance of somatic mutations across matched peripheral blood, Pap smears, and tumor tissues (363-gene Panel).

Supplementary Figure 2 Somatic mutation heat map of peripheral blood, Pap smear and tissue (16-gene Panel). Note: On the right, the gene mutation frequencies in the tissues are sorted from high to low; the bottom, from left to right, are classified by plasma, Pap smears and tissue samples; in the middle, the gene mutations and mutation frequencies in the three samples; legend: Different colors represent different degrees of mutation frequency.
