# Supplementary material for: Evaluation of cell-free DNA from papanicolaou smears and peripheral blood to detect endometrial cancer
Source: Front Oncol. 2025 Aug 11;15:1570938. doi: 10.3389/fonc.2025.1570938 (PMC12375455; doi:10.3389/fonc.2025.1570938)
Supplement: Supplementary Table 1 — Clinical and mutation information of patients with consistent mutation sites in Pap smear and tissue (16-gene Panel). FIGO, International Federation of Gynecology and Obstetrics; EIN, endometrial intraepithelial neoplasia; EEC, endometrioid endometrial carcinoma; CCC, clear cell carcinoma; UPSC, uterine papillary serous carcinoma; AF, allelic frequency of mutation detected. [file DataSheet1.docx]

**Supplementary table 1 Clinical and mutation information of patients with consistent mutation sites in Pap smear and tissue (16-gene Panel)**

| **Pap smear number** | **FIGO stage** | **Histology** | **Gene** | **DNA variant** | **Protein variant** | **Tissue AF （%）** | **Plasm AF （%）** |
| --- | --- | --- | --- | --- | --- | --- | --- |
| C1701835 | IA | G1EEC | *PIK3CA* | c.3140A>G | p.H1047R | 24.8 | 0.08 |
| C1701781 | IA | G1EEC | *PTEN* | c.388C>G | p.R130G | 82.2 | 0.20 |
| C1701935 | IA | G3EEC | *PTEN* | c.79T>G | p.Y27D | 16.1 | 5.52 |
|  |  |  | *PTEN* | c.388C>G | p.R130G | 66.3 | 16.16 |
|  |  |  | *PIK3CA* | c.1633G>A | p.E545K | 44.1 | 6.17 |
| C1701945 | IIIB | G3EEC | *PTEN* | c.83T>G | p.I28S | 42.4 | 4.74 |
|  |  |  | *ARID1A* | c.6806C>A | p.S2269X | 5.5 | 0.18 |
|  |  |  | *TP53* | c.542G>A | p.R181H | 38.1 | 3.49 |
| C1701779 | IA | G1EEC | *PTEN* | c.340G>T | p.E114X | 52.3 | 1.04 |
| C1701795 | - | EIN | *KMT2D* | c.7478G>A | p.G2493E | 3.2 | 0.05 |
|  |  |  | *CTNNB1* | c.94G>A | p.D32N | 12.2 | 0.07 |
| C1801074 | IIIC | CCC | *FBXW7* | c.1436G>A | p.R479Q | 32.0 | 0.92 |
| C1701852 | IIIB | G3EEC | *KMT2D* | c.11105G>C | p.G3702A | 4.7 | 0.07 |
| C1801368 | IA | G1EEC | *PTEN* | c.521A>G | p.Y174C | 21.9 | 3.36 |
|  |  |  | *KRAS* | c.335T>C | p.V112A | 26.4 | 5.17 |
|  |  |  | *KMT2D* | c.15797G>A | p.R5266H | 14.4 | 3.86 |
|  |  |  | *KMT2D* | c.15061C>T | p.R5021X | 27.9 | 5.15 |
|  |  |  | *KMT2D* | c.2948G>A | p.S983N | 29.0 | 5.64 |
|  |  |  | *KMT2D* | c.2747C>T | p.P916L | 26.8 | 3.78 |
|  |  |  | *ARID1A* | c.5533C>T | p.R1845W | 26.4 | 4.95 |
|  |  |  | *ARID1A* | c.5836G>T | p.A1946S | 31.4 | 5.01 |
|  |  |  | *CTCF* | c.976G>T | p.D326Y | 24.2 | 5.66 |
|  |  |  | *NF1* | c.3130T>C | p.Y1044H | 29.2 | 2.74 |
|  |  |  | *NF1* | c.4046C>T | p.A1349V | 19.5 | 4.90 |
|  |  |  | *NF1* | c.5509G>A | p.D1837N | 34.8 | 4.18 |
|  |  |  | *ARHGAP35* | c.1672A>G | p.T558A | 29.2 | 4.05 |
|  |  |  | *PIK3CA* | c.333G>T | p.K111N | 18.7 | 4.08 |
|  |  |  | *PIK3CA* | c.3141T>A | p.H1047Q | 31.5 | 4.90 |
|  |  |  | *CTNNB1* | c.569G>A | p.R190H | 29.7 | 5.16 |
|  |  |  | *FBXW7* | c.1393C>T | p.R465C | 13.1 | 3.30 |
|  |  |  | *KDR* | c.1622G>T | p.R541M | 28.7 | 4.00 |
| C1701841 | - | EIN | *PIK3CA* | c.1633G>A | p.E545K | 40.1 | 0.16 |
| C1701933 | - | EIN | *PIK3CA* | c.3140A>T | p.H1047L | 10.1 | 0.36 |
|  |  |  | *CTNNB1* | c.110C>T | p.S37F | 21.1 | 0.75 |
| C1701947 | IA | G1EEC | *FGFR2* | c.755C>G | p.S252W | 34.3 | 0.07 |
|  |  |  | *TP53* | c.743G>A | p.R248Q | 24.8 | 0.18 |
| C1701939 | IB | G2EEC | *PTEN* | c.388C>G | p.R130G | 49.5 | 1.40 |
|  |  |  | *PTEN* | c.389G>A | p.R130Q | 24.8 | 0.76 |
| C1701787 | IA | G2EEC | *PTEN* | c.253+1G>T | c.253+1G>T | 28.0 | 0.75 |
|  |  |  | *PTEN* | c.389G>T | p.R130L | 59.8 | 0.11 |
|  |  |  | *KRAS* | c.35G>A | p.G12D | 42.3 | 1.34 |
|  |  |  | *PIK3CA* | c.1359_1361delAGA | p.E453delE | 76.4 | 0.11 |
|  |  |  | *PIK3CA* | c.1636C>G | p.Q546E | 24.3 | 1.14 |
|  |  |  | *CTNNB1* | c.101G>T | p.G34V | 37.7 | 1.10 |
|  |  |  | *FBXW7* | c.1513C>T | p.R505C | 46.9 | 0.09 |
| C1701846 | - | EIN | *FGFR2* | c.755C>G | p.S252W | 21.8 | 0.21 |
| C1701790 | IA | G2EEC | *PIK3CA* | c.3143A>G | p.H1048R | 14.2 | 0.87 |
|  |  |  | *PIK3R1* | c.1744_1745+1delATG | c.1744_1745+1delATG | 12.4 | 0.12 |
| C1701928 | IA | G1EEC | *PTEN* | c.593T>A | p.M198K | 83.6 | 0.32 |
| C1701810 | IA | G1EEC | *PIK3R1* | c.1678G>T | p.D560Y | 23.3 | 0.13 |
| C1701769 | II | G3EEC | *FGFR2* | c.1144T>C | p.C382R | 25.7 | 0.12 |
|  |  |  | *PTEN* | c.389G>A | p.R130Q | 3.5 | 14.55 |
|  |  |  | *KMT2D* | c.3511G>A | p.E1171K | 28.7 | 16.26 |
|  |  |  | *NF1* | c.6787C>T | p.Q2263X | 23.8 | 14.36 |
|  |  |  | *ARHGAP35* | c.4335delC | p.G1446Afs | 47.3 | 0.24 |
| C1701796 | IA | G2EEC | *PTEN* | c.377C>A | p.A126D | 50.8 | 0.16 |
|  |  |  | *CTNNB1* | c.97T>C | p.S33P | 24.8 | 1.75 |
| C1701823 | IIIC | G2EEC | *ARID1A* | c.4494G>A | p.W1498X | 16.5 | 0.48 |
|  |  |  | *ARID1A* | c.5164C>T | p.R1722X | 3.2 | 0.25 |
| C1701808 | IA | G1EEC | *KRAS* | c.34G>T | p.G12C | 5.8 | 0.64 |
|  |  |  | *PIK3CA* | c.1624G>A | p.E542K | 31.9 | 0.71 |
| C1701792 | IA | G2EEC | *PTEN* | c.369C>G | p.H123Q | 38.6 | 0.15 |
|  |  |  | *PIK3R1* | c.1690A>G | p.N564D | 33.1 | 4.71 |
| C1701756 | - | EIN | *PTEN* | c.388C>T | p.R130X | 76.0 | 0.32 |
|  |  |  | *CTNNB1* | c.122C>T | p.T41I | 38.8 | 0.37 |
| C1701888 | IA | G2EEC | *PIK3R1* | c.1425+1G>A | c.1425+1G>A | 78.6 | 35.69 |
| C1701944 | IA | G1EEC | *PTEN* | c.389G>A | p.R130Q | 7.4 | 0.35 |
|  |  |  | *PIK3CA* | c.1035T>A | p.N345K | 15.4 | 0.60 |
|  |  |  | *CTNNB1* | c.95A>T | p.D32V | 14 | 0.71 |
| C1701799 | IB | G1EEC | *ARID5B* | c.3515C>T | p.A1172V | 10.6 | 1.35 |
|  |  |  | *PTEN* | c.44G>A | p.R15K | 8.9 | 1.57 |
|  |  |  | *PTEN* | c.562T>G | p.Y188D | 7.7 | 0.69 |
|  |  |  | *KMT2D* | c.8681C>A | p.P2894Q | 12.4 | 1.67 |
|  |  |  | *KMT2D* | c.3938G>A | p.R1313Q | 10.9 | 0.60 |
|  |  |  | *CTCF* | c.1369C>T | p.R457X | 12.7 | 1.45 |
|  |  |  | *NF1* | c.532G>T | p.E178X | 13.1 | 2.09 |
|  |  |  | *NF1* | c.3346G>T | p.D1116Y | 5.6 | 0.85 |
|  |  |  | *NF1* | c.7379C>A | p.S2460X | 16.1 | 0.93 |
|  |  |  | *NF1* | c.7915C>A | p.L2639I | 12.0 | 0.56 |
|  |  |  | *ARHGAP35* | c.1420A>C | p.M474L | 9.6 | 1.21 |
|  |  |  | *PIK3CA* | c.3129G>T | p.M1043I | 12.2 | 0.98 |
|  |  |  | *CTNNB1* | c.634C>T | p.R212C | 13.9 | 1.68 |
|  |  |  | *CTNNB1* | c.1604G>A | p.R535Q | 12.9 | 1.85 |
|  |  |  | *KDR* | c.2884C>T | p.R962C | 12.6 | 1.29 |
|  |  |  | *KDR* | c.2795G>T | p.R932I | 9.2 | 0.83 |
|  |  |  | *KDR* | c.2174G>A | p.R725H | 11.7 | 0.64 |
|  |  |  | *KDR* | c.345T>G | p.Y115X | 10.6 | 0.70 |
|  |  |  | *NOTCH1* | c.5787G>T | p.E1929D | 9.0 | 2.05 |
| C1701915 | IA | G1EEC | *PTEN* | c.697C>T | p.R233X | 52.3 | 0.19 |
| C1701819 | - | EIN | *PIK3CA* | c.1624G>A | p.E542K | 25.8 | 0.42 |
|  |  |  | *CTNNB1* | c.110C>G | p.S37C | 26.1 | 0.06 |
| C1701784 | IVB | UPSC | *TP53* | c.376-1G>A | c.376-1G>A | 36.0 | 0.74 |
|  |  |  | *PIK3R1* | c.1364A>C | p.Q455P | 36.7 | 1.08 |
| C1701793 | IA | G1EEC | *PTEN* | c.388C>G | p.R130G | 28.6 | 0.15 |
|  |  |  | *PIK3CA* | c.1633G>A | p.E545K | 34.3 | 0.36 |
| C1701854 | IA | CCC | *PTEN* | c.800delA | p.K267Rfs | 30.4 | 0.53 |
|  |  |  | *KMT2D* | c.3704delG | p.G1235Vfs | 31.7 | 0.26 |
|  |  |  | *CTCF* | c.1369C>T | p.R457X | 31.3 | 13.24 |
|  |  |  | *TP53* | c.855G>T | p.E285D | 41.4 | 13.34 |
|  |  |  | *PIK3R1* | c.1746-1G>C | c.1746-1G>C | 42.7 | 14.36 |
| C1701824 | IA | UPSC+  G2EEC | *TP53* | c.797G>A | p.G266E | 98.0 | 20.32 |
| C1701851 | IA | G2EEC | *PTEN* | c.697C>T | p.R233X | 34.1 | 0.38 |
|  |  |  | *KRAS* | c.35G>T | p.G12V | 61.4 | 0.43 |
| C1701751 | IIIC | CCC+  G2EEC | *KRAS* | c.35G>T | p.G12V | 51.1 | 0.09 |
|  |  |  | *PIK3R1* | c.1690A>G | p.N564D | 51.4 | 2.79 |
| C1701817 | - | EIN | *PIK3CA* | c.3197C>T | p.A1066V | 22.4 | 0.09 |
| C1701750 | IA | G2EEC | *PTEN* | c.100_101delinsTT | p.A34F | 20.4 | 0.21 |
| C1701831 | IVB | G3EEC | *PIK3CA* | c.1258T>C | p.C420R | 55.5 | 1.13 |
| C1701856 | - | EIN | *PTEN* | c.518G>A | p.R173H | 20.5 | 0.14 |
|  |  |  | *PTEN* | c.633_634+2delCAGT | c.633_634+2delCAGT | 14.0 | 0.08 |
|  |  |  | *PIK3CA* | c.1633G>A | p.E545K | 21.8 | 0.14 |
| C1701920 | IB | G3EEC | *PIK3R1* | c.1734_1735delinsTTACATCA | p.Q579delinsYIK | 66.8 | 0.10 |
| C1701775 | IB | G1EEC | *PIK3CA* | c.3140A>G | p.H1047R | 5.4 | 2.27 |
|  |  |  | *CTNNB1* | c.97T>C | p.S33P | 4.3 | 0.32 |
| C1701818 | IA | G1EEC | *PTEN* | c.389G>A | p.R130Q | 43.8 | 0.09 |
|  |  |  | *CTNNB1* | c.101G>T | p.G34V | 35.1 | 0.23 |
| C1701755 | IIIC | G2EEC | *ARID5B* | c.2989C>A | p.L997M | 38.9 | 7.83 |
|  |  |  | *PTEN* | c.389G>A | p.R130Q | 39.8 | 0.13 |
|  |  |  | *KMT2D* | c.12143C>T | p.P4048L | 36.8 | 1.14 |
|  |  |  | *ARID1A* | c.6420delC | p.F2141Sfs | 43.5 | 0.13 |
|  |  |  | *CTNNB1* | c.122C>T | p.T41I | 39.8 | 0.09 |
| C1701930 | - | EIN | *PIK3CA* | c.3140A>G | p.H1047R | 27.4 | 0.05 |
|  |  |  | *CTNNB1* | c.95A>T | p.D32V | 23.5 | 0.30 |
| C1701900 | IA | G1EEC | *KMT2D* | c.15845G>A | p.R5282Q | 18.9 | 0.14 |
| C1701934 | - | EIN | *PIK3CA* | c.3140A>G | p.H1047R | 8.9 | 0.10 |
| C1701847 | IIIB | G2EEC | *KRAS* | c.35G>T | p.G12V | 50.3 | 1.90 |
|  |  |  | *ARID1A* | c.1240G>T | p.G414X | 37.3 | 2.32 |
|  |  |  | *NOTCH1* | c.1220delC | p.P407Rfs | 46.7 | 0.06 |
| C1701765 | IIIC | G3EEC | *FGFR2* | c.1990C>T | p.R664W | 47.6 | 0.09 |
|  |  |  | *ARID1A* | c.4036C>T | p.Q1346X | 25.4 | 0.12 |
|  |  |  | *NF1* | c.3176A>G | p.D1059G | 24.9 | 19.31 |
| C1701804 | IA | G2EEC | *PTEN* | c.388C>G | p.R130G | 43.6 | 7.36 |
|  |  |  | *ARID1A* | c.4332_4338delGCGCCGA | p.E1444Dfs | 40.2 | 0.22 |
|  |  |  | *CTCF* | c.1117C>A | p.H373N | 37.5 | 7.98 |
| C1701837 | IA | G2EEC | *PIK3CA* | c.2128G>A | p.E710K | 5.0 | 0.25 |

Note：FIGO, International Federation of Gynecology and Obstetrics; EIN, endometrial intraepithelial neoplasia; EEC, endometrioid endometrial carcinoma; CCC, clear cell carcinoma; UPSC, uterine papillary serous carcinoma；AF, allelic frequency of mutation detected

Supplementary figure 1 Concordance of somatic mutations across matched peripheral blood, Pap smears, and tumor tissues (363-gene Panel)

**
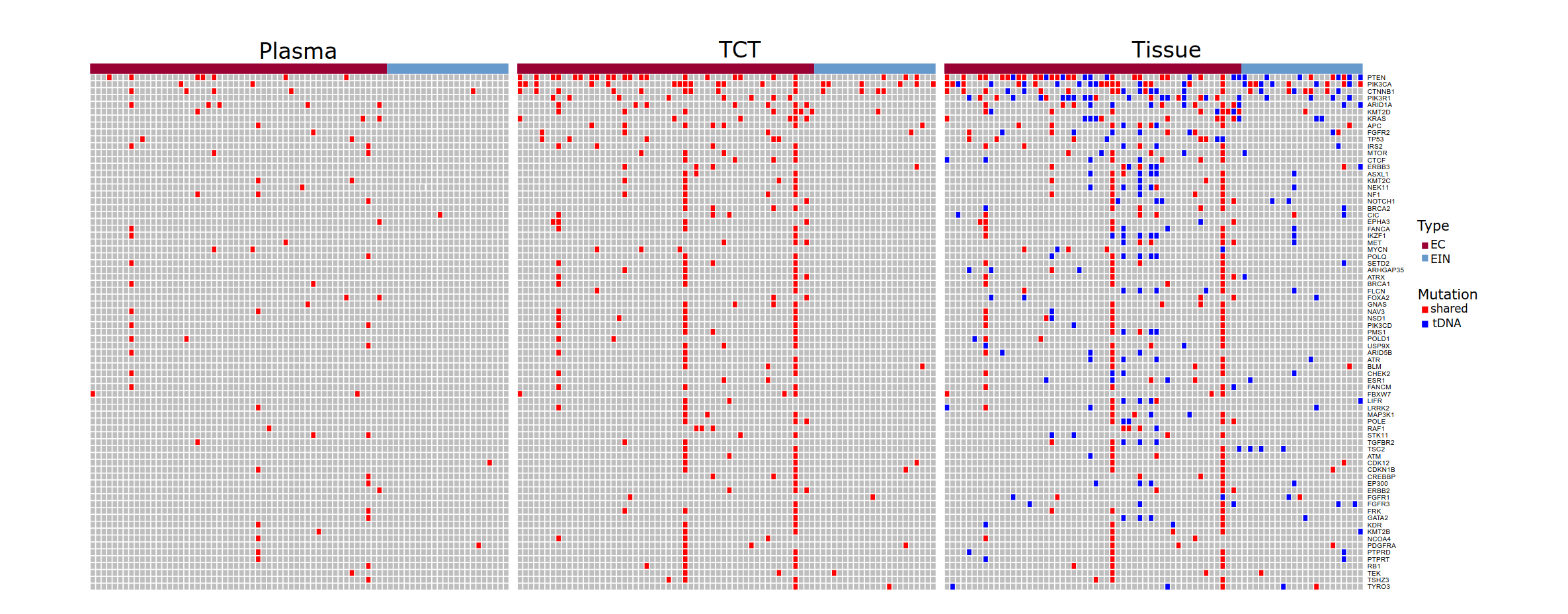
**

Supplementary figure 2 Somatic mutation heat map of peripheral blood, Pap smear and tissue (16-gene Panel)


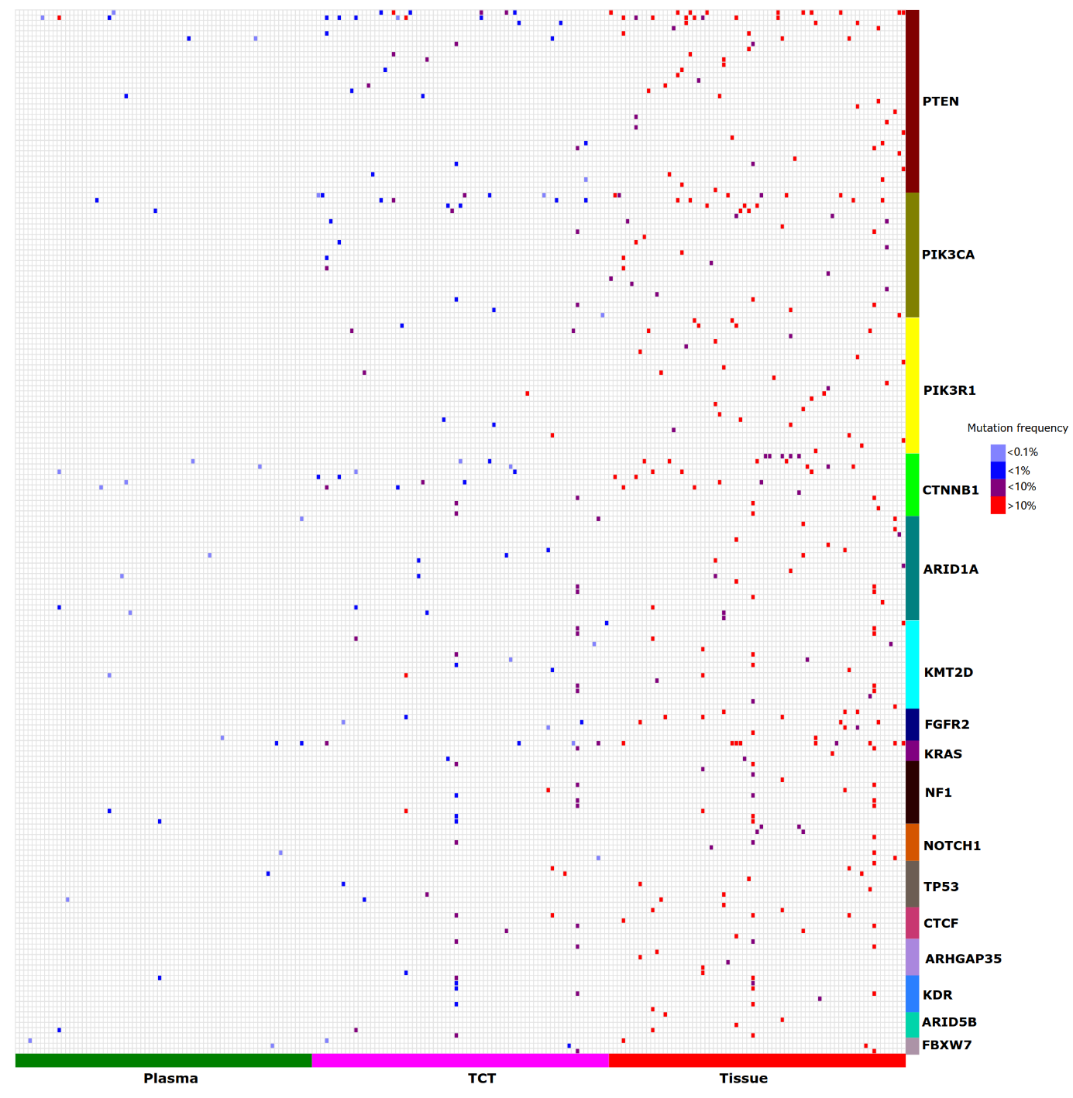
**
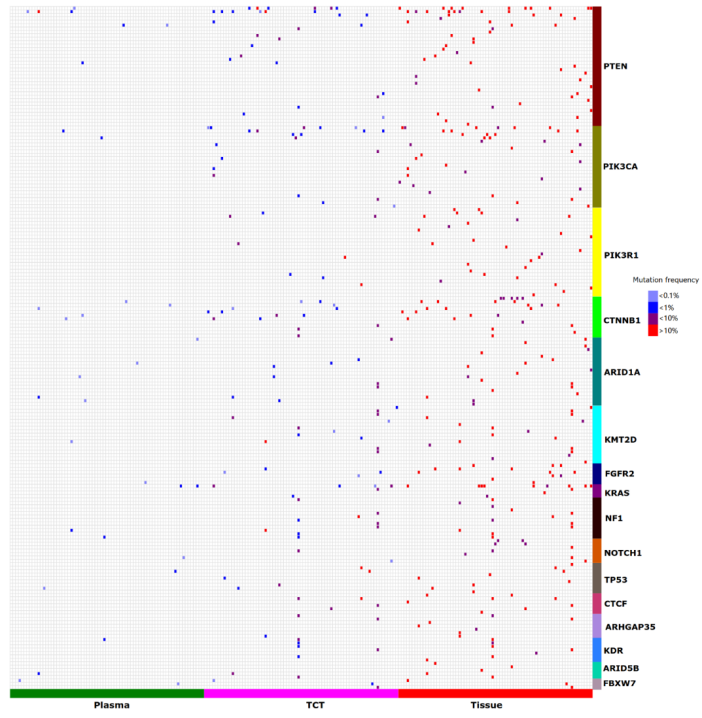
**

Note: On the right, the gene mutation frequencies in the tissues are sorted from high to low; the bottom, from left to right, are classified by plasma, Pap smears and tissue samples; in the middle, the gene mutations and mutation frequencies in the three samples; legend: Different colors represent different degrees of mutation frequency
